# The Tripartite Regulatory Framework of the Skin Extracellular Microenvironment and Related Anti-aging Strategies

**DOI:** 10.2174/0113895575436479260306062725

**Published:** 2026-04-20

**Authors:** Liangsen Zhao, Yong Liao

**Affiliations:** 1 Department of Medicine, Bloomage Biotechnology Corporation Limited, Beijing 100000, China

**Keywords:** Skin microenvironment, aging, hydration, nutrition, signaling, anti-aging strategies

## Abstract

This review proposes a tripartite regulatory framework for the Skin Extracellular Microenvironment (SEM), which the authors conceptualize as three cross-talked functional domains: hydration, nutritional, and signaling microenvironments. It systematically elaborates on the core functional architectures of each component—for instance, Hyaluronic Acid (HA) as the foundational element of the hydration microenvironment, collagen-based transport pathways in the nutritional microenvironment, and multi-axis signal networks (inflammatory, biochemical, mechanical) in the signaling microenvironment. Based on this, age-related dynamic alterations within each domain were further delineated: the hydration microenvironment undergoes progressive HA degradation and reduced biosynthesis, leading to decreased skin water retention, increased transepidermal water loss, and clinical manifestations like dryness and fine lines; the nutritional microenvironment exhibits microvascular degeneration, disrupted nutrient transport, and advanced glycation end-product accumulation, impairing extracellular matrix metabolic homeostasis; the signaling microenvironment suffers from chronic inflammation, TGF-β/Smad pathway imbalance, and mechanical signaling dysfunction, triggering a cascade of “inflammatory activation–uncontrolled degradation–mechanical disruption.” Cross-regulatory interactions among the three microenvironments (*e.g.*, hydration-signaling coupling *via* TGF-β/HAS2) are summarized to highlight their integrated regulatory nature. Additionally, targeted anti-aging strategies are discussed: exogenous HA supplementation and endogenous activation for the hydration microenvironment, metabolic substrate supply (*e.g.*, proline, trace elements) for the nutritional microenvironment, and multi-pathway modulation (*e.g.*, anti-inflammatory agents, platelet-rich plasma, collagen stimulants) for the signaling microenvironment, with particular attention to the synergistic effects observed in combination approaches such as mesotherapy-based cocktails and exosome therapy. Overall, this framework integrates previously fragmented SEM research and provides a coherent theoretical basis for evidence-based clinical anti-aging strategies and future precision interventions targeting skin aging.

## 1 INTRODUCTION

**Fig. (1):**
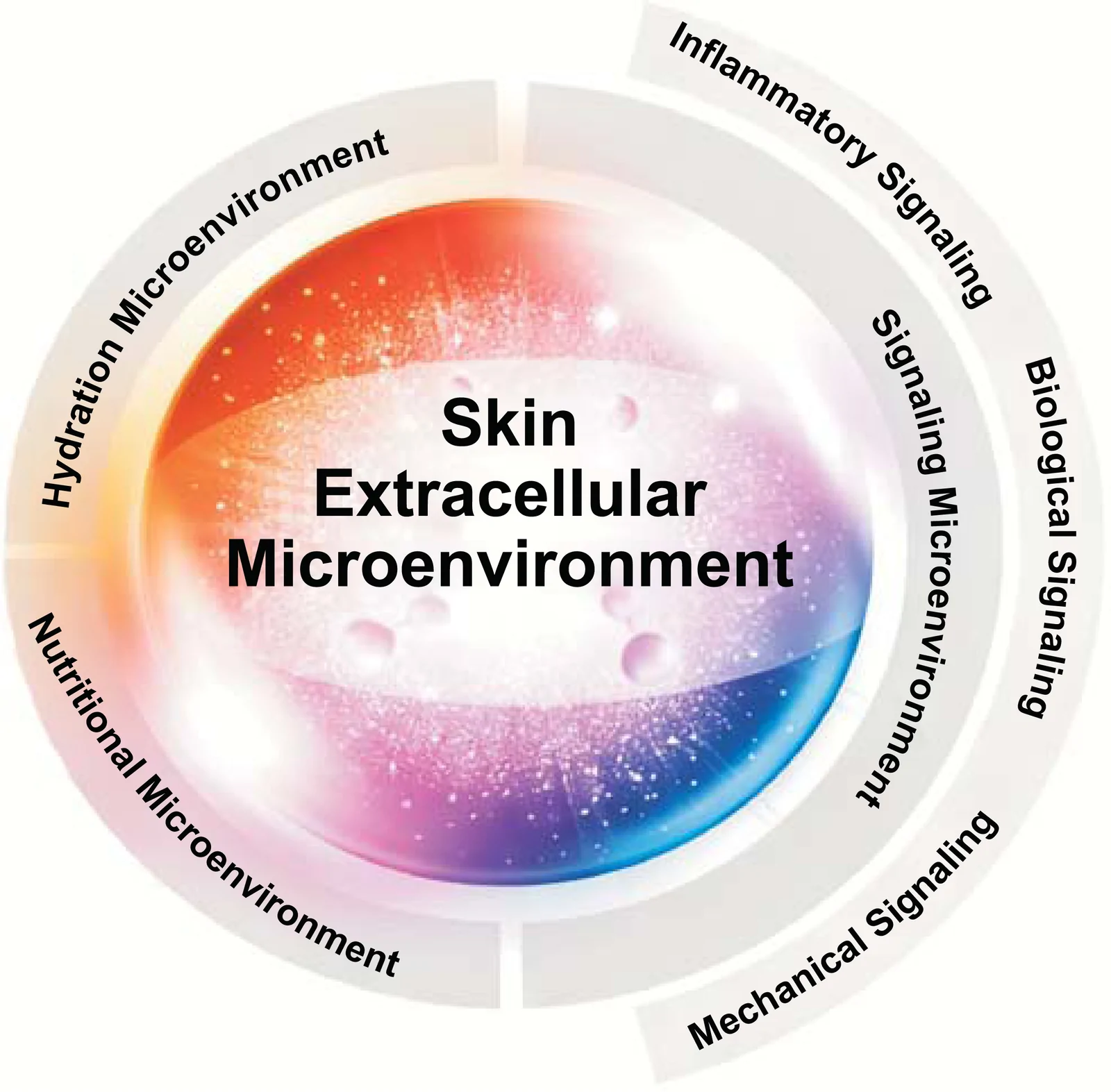
Schematic diagram of the components and functional architecture of the SEM. **Note: Core framework:** Illustrates the tripartite classification of the SEM into three interconnected functional domains—hydration microenvironment, nutritional microenvironment, and signaling microenvironment—forming a holistic regulatory network for skin homeostasis. **Component details:** Hydration microenvironment: Centered on hyaluronic acid and glycosaminoglycans, responsible for maintaining skin water retention and ECM elastic buffering. Nutritional microenvironment: Composed of collagen-based transport channels, amino acids, vitamins, and trace elements, providing metabolic substrates for dermal/epidermal cells. Signaling microenvironment: Integrates three sub-axes (inflammatory, biochemical, mechanical) to mediate cell regulation *via* molecular crosstalk/crosslink. **Functional significance:** Visualizes the structural basis of each microenvironment and its synergistic relationships, laying the foundation for understanding SEM-mediated skin physiological regulation(Image source: Designed and produced by the author's team, with copyright owned by the author).

**Fig. (2):**
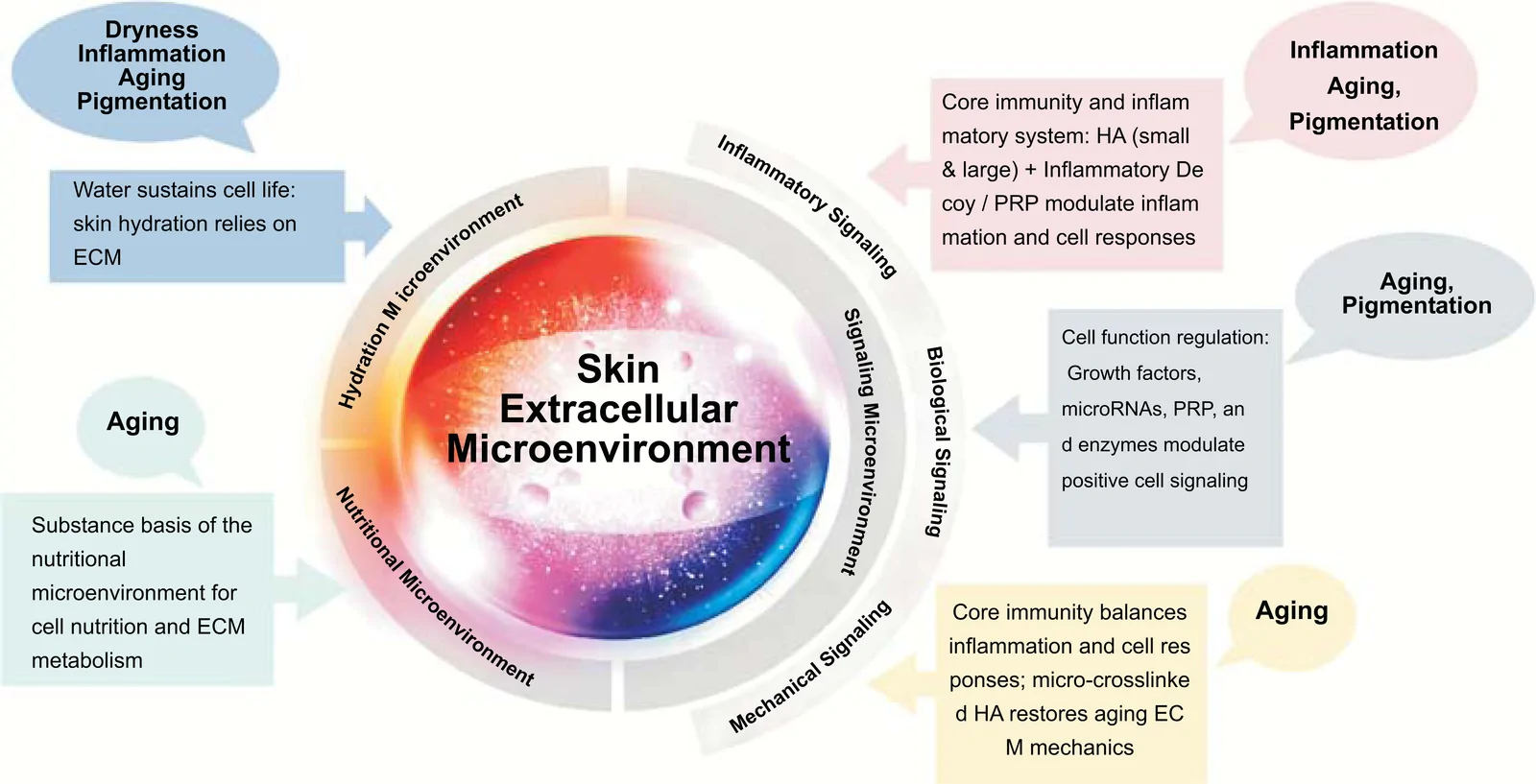
Schematic diagram of age-related pathological mechanisms of the SEM and targeted regulatory pathways. **Note: Pathological cascade:** Takes the SEM as the core, depicting the characteristic degenerative changes of the three microenvironments during skin aging and their association with clinical phenotypes: Hydration microenvironment: HA degradation/synthesis imbalance leads to dryness, increased transepidermal water loss, and fine lines. Nutritional microenvironment: Microvascular degeneration and collagen rigidity cause nutrient transport disorders, exacerbating the ECM metabolic imbalance. Signaling microenvironment: Chronic inflammation, TGF-β/Smad pathway dysfunction, and impaired mechanical sensing trigger “inflammatory activation–uncontrolled degradation–mechanical disruption,” contributing to skin laxity, pigmentation, and aging. **Regulatory strategies:** Highlights key interventions targeting each microenvironment, including HA supplementation (hydration), nutrient precursor supply (nutrition), and multi-pathway modulation *via* PRP, growth factors, and micro-crosslinked HA (signaling), which collectively regulate immunity, inflammation, and cell function to repair aged ECM (Image source: Designed and produced by the author's team, with copyright owned by the author).

As the largest organ of the human body, the skin relies on the precise regulation of the extracellular microenvironment to preserve the structural and functional integrity of its cellular components and Extracellular Matrix (ECM) structure. Although prior studies have generated substantial evidence mostly by focusing on individual ECM components or discrete signaling pathways, the extracellular microenvironment has rarely been integrated as an overall dynamically regulated system. Herein, a “*Three-Dimensional Theory of the Skin Extracellular Microenvironment (SEM)*” was proposed (Fig. **1**), which classifies the SEM into three functional domains: the hydration microenvironment, the nutritional microenvironment, and the signaling microenvironment. From our perspective, this framework allows a more coherent interpretation of how coordinated microenvironmental alterations accompany skin aging, especially the ECM, rather than viewing these changes as isolated events. This framework preliminarily delineates the dynamic alterations in these microenvironments during the aging of skin cells and the ECM, and puts forward multidimensional anti-aging strategies targeting the SEM, with a specific focus on mesotherapy-based personalized interventions. The objective of this review is therefore not to introduce new biological entities, but to integrate existing skin SEM-related findings into a unified conceptual model, thereby supporting more evidence-based and theoretical framework-supported clinical approaches for skin anti-aging (Fig. **2**).

## 2 THE THREE-DIMENSIONAL FUNCTIONAL ARCHITECTURE OF THE SKIN EXTRACELLULAR MICROENVIRONMENT

### 2.1 Hydration Microenvironment

The hydration microenvironment serves as the core foundation for sustaining the physiological functions of skin cells and is primarily formed by Hyaluronic Acid (HA), a molecule with potent water-binding capacity. HA exhibits a molecular weight ranging from several kilodaltons to several hundred kilodaltons, with each molecule capable of binding up to 1,000 times its own weight in water [1]. Through electrostatic interactions, HA assembles into an amorphous gel-like matrix that fills interfibrillar spaces, acting as the principal “hydrated matrix” of the ECM [2]. This unique structural feature explains how HA translates its physicochemical water-binding capacity into a functional regulator of skin hydration: in the papillary dermis, HA forms a loose network with fine collagen fibers, facilitating the diffusion of nutrients to the avascular epidermis [3]; in the reticular dermis, HA modulates hydration within interfibrillar gaps, regulating the reversible elastic deformation of elastic fibers [4]. Other Glycosaminoglycans (GAGs), including Dermatan Sulfate (DS) and chondroitin sulfate, stabilize the hydration microenvironment through the modification of proteoglycans such as decorin [5]. DS-proteoglycan complexes (*e.g.* decorin) can bind Transforming Growth Factor-β (TGF-β), suppressing the TGF-β/Smad pathway and indirectly regulating HA synthase 2 (HAS2) activity, thereby forming a cross-regulatory “hydration–signaling” loop [6]. This dynamic equilibrium, inferred from these interactions, equips the skin with the capacity to buffer external mechanical stress while preserving hydration, thus preventing structural and functional disruption of the ECM induced by overhydration or dehydration (Table **1**) [7].

### 2.2 Nutritional Microenvironment

The nutritional microenvironment provides metabolic substrates to both dermal and epidermal cells and includes structural transport pathways as well as functional nutrients [8]. Type I collagen, which constitutes over 80% of the total dermal protein content, forms cross-linked bundles that impart mechanical scaffolding while establishing channels for nutrient diffusion, with nutrients supplied by the local terminal microcirculatory system [9]. In the papillary dermis, fine collagen fibers and abundant GAGs form a porous network. This structure facilitates the diffusion of amino acids, glucose, trace elements, and minerals to the avascular epidermis [9]. In the reticular dermis, thick collagen and elastic fibers propel interstitial fluid flow through periodic deformation. This process enhances the exchange of nutrients and metabolites between nearby blood and lymphatic vessels [10]. Functional glycoconjugates contribute to the regulation of nutrient homeostasis through dual mechanisms: HA degradation products (oligosaccharides) activate the mTORC1 pathway *via* CD44 receptors, thereby regulating amino acid transport and protein synthesis [11]. Proteoglycans such as perlecan bind growth factors, including Insulin-like Growth Factor 1 (IGF-1) and Fibroblast Growth Factor 2 (FGF-2), forming controlled-release reservoirs for spatiotemporal signaling delivery [8]. Together, these structure-function relationships support ongoing extracellular matrix synthesis and remodeling by fibroblasts and keratinocytes, which are essential for maintaining metabolic homeostasis in the skin (Table **1**) [10].

### 2.3 Signaling Microenvironment

The signaling microenvironment serves as the central functional axis of signal-mediated cell regulation. It integrates inflammatory, biochemical, and mechanical signals that interact through molecular crosstalk and cross-linking to form a dynamically and effectively regulatory network [12]. Inflammation, a process involving injury, defense, and repair, is mediated by inflammatory triggers, receptors, signaling molecules, and effectors, including ECM damage associated with skin aging. The Toll-like receptor (TLR)–NF-κB axis plays a pivotal role in the recognition of ECM damage. For example, HA degradation products (*e.g.*, low-molecular-weight HA and HA oligosaccharides) are recognized by TLR4, which then triggers inflammatory cascades, induces the expression of matrix metalloproteinases (MMPs), and initiates a “damage–immune recognition–inflammation–degradation” pathway [13]. Based on existing evidence, the authors consider that homeostatic inflammation plays a distinct role from chronic inflammation and contributes to ECM remodeling by regulating the balance between MMPs and their inhibitors (tissue inhibitors of metalloproteinases, TIMPs), thereby facilitating collagen turnover during the early phase of ECM healing.

The biochemical signaling axis centers on two core pathways: the TGF-β/Smad and PI3K/Akt pathways. Signals—including growth factors, messenger RNA, and enzymes—mediate intercellular communication and thereby modulate cellular functions (*e.g.*, the synthesis of ECM components), ultimately regulating ECM integrity [14]. Additionally, fibroblast-derived exosomes containing miR-21 can suppress MMP-3 expression in neighboring fibroblasts, thereby reducing collagen degradation and contributing to ECM homeostasis [15]. From our perspective, the functional heterogeneity of dermal fibroblasts further highlights the importance of precise biochemical regulation, as CD34+PDGFRα+ subpopulations in the papillary dermis predominantly express COL3A1, while CD26+ subpopulations in the reticular dermis are responsible for elastic fiber synthesis—both of which are precisely regulated by the biochemical signaling axis (*e.g.*, epidermal growth factor and fibroblast growth factor) [16-18].

The mechanical signaling axis operates through the “SEM mechanics–cell mechanosensing–gene expression” cascade. Collagen structure and crosslinking dictate SEM stiffness, which fibroblasts sense *via* β1 integrins, thereby triggering YAP/TAZ nuclear translocation and downstream ECM gene expression [19]. For example, fibroblasts interact with collagen *via* integrins (*e.g.*, α2β1), forming focal adhesions that activate FAK/PI3K signaling, thereby regulating COL1A1 transcription and secretion. This mechanical-signaling feedback loop is a key adaptive mechanism, enabling the ECM to adapt its structural composition in response to mechanical stimuli (*e.g.*, tension, pressure), thus preserving tissue mechanical homeostasis. As described in “Niche stiffness regulates stem cell aging”, modulating matrix stiffness through biomechanics approaches represents a promising strategy to mitigate skin aging [20].

These three microenvironments do not operate independently; rather, they interact synergistically to form an integrated regulatory network (Table **1**). For instance, HA in the hydration microenvironment binds growth factors like FGF and collaborates with the signaling microenvironment to regulate target cell function [21, 22]. Collagen precursors (*e.g.*, proline) in the nutritional microenvironment serve both as substrates and modulators of signaling efficiency *via* the mTOR pathway. Taken together, the TGF-β pathway occupies a central coordinating position, as it simultaneously regulates HA and collagen synthesis, thereby influencing both hydration and mechanical properties of the SEM. This synergistic mechanism enables SEM to respond systemically to internal and external stimuli and changes, thereby maintaining skin structural and functional homeostasis (Supplementary Figure **S1**) [23, 24].

## 3 EXTRACELLULAR MICROENVIRONMENT IN SKIN AGING

### 3.1 Degeneration of the Hydration Microenvironment

With skin aging, HA—the core component of the hydration microenvironment—undergoes progressive degradation and reduced biosynthesis. Enhanced Hyaluronidase (HYAL) activity cleaves HA chains, lowering their average molecular weight from high-molecular-weight forms in youth to significantly reduced levels in aged skin, thereby impairing water-binding capacity [21]. Concurrently, the expression of Hyaluronan Synthases (HAS) is downregulated, particularly HAS2, whose promoter exhibits increased methylation, resulting in a sustained reduction in endogenous HA production [25]. Taken together, this coupled process of accelerated HA degradation and insufficient biosynthesis, rather than either change alone, underlies the progressive failure of hydration maintenance in the aging extracellular microenvironment, clinically manifesting as increased transepidermal water loss (TEWL), reduced water content in both epidermis and dermis, and features such as dryness, flaking, and fine lines [26]. Alterations in the sulfation pattern of GAGs further destabilize the hydration microenvironment. The ratio of chondroitin sulfate to dermatan sulfate becomes markedly dysregulated in aged skin, with an overall decline in sulfation diminishing the electrostatic affinity of GAGs for water molecules [27]. This qualitative alteration in GAG composition weakens the gel-like organization of the ECM, a conclusion supported by electron microscopy showing increased ECM porosity and the conversion of a previously uniform gel matrix into a relatively “dry network” structure [28]. Such structural changes not only reduce water retention but also impede nutrient diffusion, thereby reinforcing a self-perpetuating cycle of “hydration deficiency-nutritional impairment”.

### 3.2 Metabolic Decline of the Nutritional Microenvironment

Aging of the nutritional microenvironment is characterized by microvascular degeneration, reduced supply of metabolic substrates, and associated imbalances in the synthesis and degradation of structural and functional components. Degenerative alterations in dermal microcirculation including terminal arterioles and venules compromise nutrient delivery to the skin tissue. The ratio of type I to type III collagen increases with age, shifting from a balanced youthful state to a rigid-dominated matrix in aged skin. This shift toward collagen I dominance alters the mechanical properties of the ECM, enhancing collagen fiber stiffness while diminishing elasticity, forming a “rigid network”. When combined with age-related degeneration of GAG-based gel matrices, these changes impair intradermal nutrient transport [29]. Spatiotemporal disruption of functional nutrient components exacerbates metabolic decline. The weakened binding between proteoglycans (*e.g.*, perlecan) and growth factors in the aged ECM reduces the effective local availability of regulators such as IGF-1 and TGF-β, thereby impairing fibroblast nutrient-sensing pathways (*e.g.*, mTOR) [30]. Additionally, the sustained accumulation of advanced glycation end-products (AGEs) induces irreversible AGE–collagen crosslinks, which activate the NF-κB signaling pathway *via* Receptor for Advanced Glycation End Products (RAGE) receptors. This AGE-driven signaling not only promotes structural stiffening but also suppresses nutrient transporter expression, disrupting cellular metabolism at both supply and uptake levels [31].

### 3.3 Dysregulation of the Signaling Microenvironment

During skin aging, the signaling microenvironment is characterized by imbalances across three major pathways, which are interpreted as a progressive cascade of “inflammatory activation–uncontrolled degradation–mechanical disruption.” The most prominent alteration resides in chronic inflammation and associated damage. Intrinsic and extrinsic factors of skin aging activate the JAK/STAT and NF-κB pathways, sustaining a persistent, low-grade inflammatory state [32]. This sterile inflammatory milieu shifts ECM turnover toward degradation, upregulating degradation enzymes (*e.g.*, MMP-1, MMP-9) while attenuating TIMP-mediated inhibition, thereby disrupting ECM homeostasis. Clinically, MMP-1 activity is significantly elevated in aged skin, accelerating the degradation of type I collagen [33]. Furthermore, inflammation impairs the function of keratinocytes and melanocytes, contributing to epidermal degradation and pigmentary abnormalities [34].

Biochemical signaling imbalances manifest as dual impairments in regulatory pathways. The expression of growth factors, messenger RNAs, and various enzymes is downregulated with aging. Additionally, the TGF-β/Smad pathway in senescent fibroblasts exhibits insufficient activation and enhanced inhibition: phosphorylation of Smad2/3 decreases, thereby weakening TGF-β–induced COL1A1 transcription [35]. Meanwhile, telomere shortening and DNA damage trigger cellular senescence, stimulating the release of Senescence-Associated Secretory Phenotypes (SASP), including Interleukin-1 (IL-1) and Tumor Necrosis Factor (TNF-α). This SASP-related cytokines amplify signaling dysfunction, as IL-1β promotes p38MAPK-mediated phosphorylation of Smad3, converting it into a transcriptional repressor [36]. Taken together, these changes converge on a net reduction in ECM gene expression, initiating a pathological cascade of “signal imbalance–synthetic failure”.

Mechanical signaling dysfunction represents a physical manifestation of signaling deterioration. ECM synthesis decreases while degradation increases, accompanied by solar elastosis and reduced crosslinking of collagen and elastic fibers. Moreover, collagen fibers transition from an orderly to a disorganized spatial arrangement, with electron microscopy demonstrating heterogeneity in fiber diameters and an imbalanced stress distribution [37]. Lysyl Oxidase (LOX) activity declines, compromising ECM stiffness and elasticity. Reduced β1 integrin membrane localization in aged fibroblasts further weakens mechanosensing, lowering their affinity for collagen and impairing cell mechanosensing. This suppresses the nuclear translocation of YAP/TAZ, thereby downregulating the transcription of ECM-related genes (*e.g.*, COL1A1, ELN) [38]. This creates a negative feedback loop between structural degradation and signaling impairment.

These microenvironmental alterations during skin aging are not isolated but intricately interconnected. HA degradation products within the hydration microenvironment activate the inflammatory axis *via* the TLR4/NF-κB pathway, upregulating MMP expression and promoting collagen catabolism, thereby disrupting mechanical and nutritional homeostasis [38]. AGEs in the nutritional microenvironment trigger inflammatory signaling *via* RAGE receptors and concurrently suppress HA synthase activity, compromising skin hydration [39, 40]. The decline in mechanical signaling, as a downstream amplifier, inhibits fibroblast-derived HA and collagen biosynthesis, further undermining hydration status, nutrient supply, and ECM mechanical integrity [41]. Collectively, these aging forms of crosstalk drive the SEM toward regulated adaptation, rather than progressive structural disorganization, which clinically manifests as skin laxity, deeper rhytides, reduced elasticity, and a dull cutaneous appearance.

## 4 EXTRACELLULAR MICROENVIRONMENT–TARGETED ANTI-AGING STRATEGIES

### 4.1 Hydration Microenvironment Strategy

Interventions targeting age-related deterioration of the hydration microenvironment employ a dual strategy: exogenous HA supplementation and endogenous HA activation (Table **2**). Non-crosslinked HA exerts its biological effects *via* multiple delivery modalities: transdermal application (topical formulations) and mesotherapy (Supplementary Figure **S1**). HA products with varying molecular weights penetrate the stratum corneum and access the dermis through minimally invasive techniques, activating CD44-mediated signaling cascades to facilitate the synthesis and regeneration of endogenous high-molecular-weight HA [42-44]. Low-crosslinked HA enhances molecular stability, directly replenishing depleted HA reserves while inducing HAS expression *via* mechanical signaling pathways. This combined effect distinguishes low-crosslinked HA from simple filler replacement, as this dual mechanism—immediate replenishment and stimulation of endogenous synthesis—enables it not only to acutely restore HA concentrations but also to sustain long-term tissue homeostasis. Compared to highly crosslinked counterparts, its lower crosslinking density ensures superior biocompatibility and favorable degradation kinetics in skin, aligning with physiological tissue repair processes. Clinical investigations on low-crosslinked HA demonstrate that it reduces TEWL and enhances hydration in facial photoaging, with high-dose intradermal injections (48 mg) delivering longer-lasting effects (up to 6 months) than low-dose regimens [45]. Its efficacy is also validated in subcutaneous fat layer injections, improving skin texture with 93.3% patient satisfaction at 3 months [46]. Furthermore, low-crosslinked HA can be integrated into combination therapeutic approaches, such as synergizing with botulinum toxin microinjections to augment hydration-associated skin quality [47]. Taken together, these clinical observations suggest that the hydration-maintenance benefit of low-crosslinked HA extends beyond short-term volume effects and is partly attributable to modulation of fibroblast mechanotransduction pathways and the related synthesis of ECM components.

### 4.2 Nutritional Microenvironment Strategy

Interventions targeting the nutritional microenvironment prioritize substrate supplementation, metabolic activation, and signaling pathway modulation (Table **2**). Supplementation with precursors (*e.g.*, proline, lysine, hydroxyproline) enhances collagen biosynthesis. Proline, as a key amino acid in the triple-helix structure of collagen, accelerates collagen production following exogenous supplementation, while lysine facilitates elastin crosslinking, improving the mechanical properties of the fiber network [48, 49]. Metabolic imbalance correction is also achieved through supplementation with trace elements and minerals [49]. Copper ions, acting as cofactors for LOX, enhance elastin crosslinking when administered locally [50, 51]; zinc inhibits MMP-1 activity, attenuating collagen degradation [52]. Vitamin D, through VDR-mediated signaling, regulates COL1A1 transcription, with tissue levels positively correlated with collagen density. Vitamin D supplementation promotes collagen gene expression [53].

Hyaluronate composite solutions (skin boosters)—which contain HA with amino acids (*e.g.*, glycine, proline, alanine)—further optimize the nutritional microenvironment. These formulations provide dual nutritional support: HA replenishes ECM components and sustains hydration, while amino acids serve as direct substrates for collagen and elastin synthesis, stimulating fibroblast activity and enhancing ECM remodeling [54, 55]. Studies demonstrate that such solutions improve nutrient diffusion in the dermal microenvironment, upregulate type I collagen expression, and enhance skin metabolic homeostasis, complementing traditional micronutrient supplementation [56, 57]. The advantage of these composite formulations lies in their coordinated support of both ECM structure and cellular metabolism, as the authors interpret their effects as converging on key enzymatic reactions and signaling pathways that help maintain metabolic activity within the nutritional microenvironment, rather than acting through isolated nutrient replacement alone.

### 4.3 Signaling Microenvironment Strategy

Interventions targeting the signaling microenvironment aim to modulate inflammation, activate biological signals, and regulate mechanical cues through multi-target synergy (Table **2**). These interventions are necessary responses to the intrinsically coupled nature of inflammatory, biochemical, and mechanical signaling during skin aging, rather than as isolated pathway corrections. Anti-inflammatory agents block aberrant signaling pathways. For instance, vitamin C—an important anti-inflammatory molecule—acts as a cofactor for prolyl hydroxylase, facilitating collagen folding while scavenging Reactive Oxygen Species (ROS) to suppress the NF-κB pathway, and also significantly reduces MMP-1 expression [58]. Tranexamic acid modulates inflammation by inhibiting plasminogen activation and its associated inflammatory cascades.

Platelet-Rich Plasma (PRP) delivers a spectrum of growth factors, *e.g.*, Platelet-Derived Growth Factor (PDGF), TGF-β1, Vascular Endothelial Growth Factor (VEGF), which activate the PI3K/Akt pathway to promote collagen synthesis, inhibit the JAK/STAT to attenuate inflammatory signaling, and enhance vascular supply to optimize the nutritional microenvironment [59]. Notably, PRP exerts potent anti-inflammatory effects by downregulating pro-inflammatory cytokines (*e.g.*, IL-6, TNF-α) and reducing neutrophil infiltration-findings supported by clinical studies demonstrating decreased skin redness and edema post-treatment [60]. It also upregulates anti-inflammatory factors, such as IL-10, balancing the local immune microenvironment to prevent chronic inflammation-induced ECM degradation [61]. The effect of PRP administration is comprehensive because it simultaneously amplifies regenerative signals while constraining SASP-driven inflammation, as shown by the finding that repeated PRP treatments increase TGF-β activity and reduce SASP factors (*e.g.*, IL-6), achieving bidirectional regulation of the signaling microenvironment [62].

Collagen stimulators, including poly-L-lactic acid (PLLA), poly-D, L-lactic acid (PDLLA), and calcium hydroxylapatite (CaHA), function as exogenous cellular stimuli. They induce fibroblast activation *via* microinflammation or sustained calcium release, leading to neocollagenesis [63-65]. Clinically, intradermal PDLLA injections significantly increase collagen density over months, with concurrent improvements in the nutritional microenvironment [66]. Soluble collagen peptides (*e.g.*, type I collagen peptides) stimulate fibroblasts *via* 'peptide sensing', thereby activating the mTOR pathway and promoting endogenous collagen synthesis [67, 68].

Mechanical signaling modulation remodels the SEM by physically reconstituting signal networks. Low-crosslinked HA administered *via* intradermal or subcutaneous fat injection increases local tissue tension, stimulating fibroblasts to upregulate collagen synthesis and improve skin elasticity [69]. These effects provide evidence that moderate mechanical reinforcement can partially restore downstream signaling competence, as high-dose regimens yield more pronounced and durable outcomes, with observed improvements in skin viscoelasticity and wrinkle reduction-validating their regulatory role in the mechanical microenvironment [45, 46, 70].

Single-pathway interventions often fail to address the complex crosstalk within the extracellular microenvironment during skin aging [71]. This limitation explains the growing clinical practice of multi-target strategies rather than single-agent or technology. Emerging mesotherapy-based cocktail therapies employ multi-component, multimodal approaches for systemic ECM remodeling [72]. For example, the “HA combined PRP injection” combination protocol integrates hydration enhancement (*via* HA), signaling modulation (*via* PRP), and mechanical activation (*via* low-crosslinked HA), delivering superior improvements in hydration status, collagen density, and elastin crosslinking compared to monotherapies [73]. HA-based cocktails (*e.g.*, kinetin-containing complexes) modulate the hydration microenvironment through multi-component synergy [74]. Beyond HA, these formulations typically incorporate amino acids, vitamins, and minerals: proline acts as a precursor for collagen biosynthesis, vitamin B5 (pantothenic acid) supports skin barrier repair, and zinc regulates HYAL activity [75]. Therefore, this “hydration-nutrition” dual replenishment strategy is considered to be a functional extension of signaling modulation, rather than a purely supportive intervention. Exosome therapy represents a novel synergistic approach with distinct advantages [76, 77]. Derived from mesenchymal stem cells, exosomes are enriched in miRNAs (*e.g.*, miR-29), growth factors (*e.g.*, VEGF, fibroblast growth factor (FGF), TGF-β), and antioxidant molecules [78, 79]. Exosome-based therapies can be considered to achieve coordinated, three-dimensional regulation across signaling, hydration, and nutritional axes. Through targeted delivery, they modulate these axes: activating fibroblasts (signaling axis), enhancing HA synthesis (hydration axis), and promoting collagen transcription (nutrition axis).

## CONCLUSION

The tripartite regulatory framework of the SEM—encompassing hydration, nutritional, and signaling axes- offers a more integrative perspective that differs from traditional single-pathway research. These functional components interact through molecular crosstalk and cross-regulatory networks to sustain skin homeostasis, and their coordinated degeneration, as supported by current evidence, appears to drive pathogenesis of skin aging (*e.g.*, impaired hydration, metabolic dysregulation, signaling dysfunction). On this basis, targeted interventions targeting individual components (*e.g.*, HA supplementation, nutrient modulation, signaling pathway regulation) can remodel the SEM, with combinatorial approaches consistently showing greater efficacy than monotherapies. Future research should prioritize three directions: microenvironmental heterogeneity, biomimetic experimental models, and precision-targeted strategies—collectively advancing the field of regenerative and anti-aging medicine.

## Figures and Tables

**Table 1:** Core characteristics of the SEM.

**Microenvironment Type**	**Core Components**	**Main Functions**	**Age-related Changes**	**Key Regulatory Pathways**	**References**
Hydration Microenvironment	HA, GAGs, such as dermatan sulfate, chondroitin sulfate	Maintain skin hydration, form a hydrated matrix, and regulate mechanical buffering capacity	Increased HA degradation and decreased synthesis (down-regulation of HAS2 expression); imbalance in GAGs sulfation pattern, reduced water-binding capacity	TGF-β/HAS2, TLR4/NF-kB (cross-regulation)	[1, 2, 5, 6, 21, 25, 27]
Nutritional Microenvironment	Amino acids (proline, lysine), Mineral salts, Vitamin, Nucleic acids	Provide metabolic substrates, construct nutrient diffusion channels, and regulate ECM synthesis and remodeling	Increased collagen degradation and decreased synthesis; Imbalance in collagen ratio (increase in Type I/Type III collagen); accumulation of AGEs leading to abnormal collagen cross-linking; decreased nutrient transport efficiency	mTOR (amino acid sensing), LOX (collagen cross-linking)	[8, 9, 11, 29, 31, 48, 49]
Signaling Microenvironment	Inflammatory signals (LMW-HA, TLR4); Biochemical signals (TGF-β, miR-21); Mechanical signals (collagen stiffness, β1 integrin)	Regulate the balance between ECM repair and degradation, control cell functions (proliferation, synthesis), and sense mechanical stimuli to adjust ECM structure adaptively.	Chronic inflammation (imbalance of MMPs/TIMPs); weakened TGF-β/Smad signaling; abnormal mechanical sensing (reduced YAP/TAZ nuclear translocation)	NF-kB (inflammation), TGF-β/Smad (biochemical), YAP/TAZ (mechanical)	[13-15, 19, 32, 35, 37]

**Table 2:** Precision anti-aging interventions and mechanisms targeting the SEM.

**Intervention Target**	**Specific Intervention Methods** **(Including Formulations/Techniques)**	**Molecular Mechanisms**	**Key Biomarker Changes**	**References**
Hydration Microenvironment	Non-crosslinked HA: Transdermal administration, microneedle, or injection	Activate CD44 receptors to promote endogenous high-molecular-weight HA synthesis.	Reduced TEWL; increased skin water content; up-regulation of HAS2 mRNA expression	[42-47, 69, 70]
	Low-crosslinked hyaluronic acid: Intradermal injection	Induce HAS expression through mechanical signals, prolonging the maintenance time of hydration.		
Nutritional Microenvironment	Amino acid, mineral salts, Vitamin, nucleic acids: Topical/oral administration/dermal injection	Serve as precursors for the collagen triple-helix structure, accelerating COL1A1 synthesis.	Increased collagen density; elevated hydroxyproline content; activation of the mTOR pathway	[48-50, 52-57]
	HA-based composite solutions (containing glycine, alanine): Dermal injection	Optimize the nutrient diffusion microenvironment and activate fibroblast metabolism.		
Signaling Microenvironment	Anti-inflammatory interventions: Vitamin C, polydeoxyribonucleotide (PDRN) and polynucleotides (PN): Dermal injection	Inhibit the NF-kB pathway, reduce MMP-1 expression, and act as a cofactor for prolyl hydroxylase to promote collagen folding.	Decreased levels of inflammatory factors (IL-6, TNF-α); enhanced TGF-β activity; up-regulation of collagen-related genes (COL1A1, ELN)	[58-68, 76-78]
	Growth factors, PRP, stromal vascular fraction (SVF), Exosome, Secretomes, coenzymes: Dermal injection	Release PDGF-BB/TGF-β1, activate the PI3K/Akt pathway, and down-regulate SASP factors such as IL-6		
	Collagen stimulants: PLLA, PDLLA, CaHA: Dermal /subcutaneous injection	Induce fibroblast activation through micro-inflammation, up-regulating COL1A1 transcription.		
	Mechanical regulation: low- crosslinked HA: Injection in the dermal and superficial fascia layer	Increase local tension, activate YAP/TAZ nuclear translocation through β1 integrin, and promote ECM gene expression.	Enhanced YAP/TAZ nuclear localization; up-regulation of ECM-related genes (COL1A1, ELN); increased collagen deposition	[37, 46, 69, 70]

## LIST OF ABBREVIATIONS

AGEs: Advanced Glycation End Products
Akt: Protein Kinase B
CaHA: Calcium Hydroxylapatite
DS: Dermatan Sulfate
ECM: Extracellular Matrix
ELN: Elastin
FAK: Focal Adhesion Kinase
FGF-2: Fibroblast Growth Factor 2
GAGs: Glycosaminoglycans
HA: Hyaluronic Acid
HAS: Hyaluronan Synthase
HAS2: Hyaluronan Synthase 2
HGF: Hepatocyte Growth Factor
HYAL: Hyaluronidase
IGF-1: Insulin-Like Growth Factor 1
IL: Interleukin
JAK: Janus Kinase
LOX: Lysyl Oxidase
MAPK: Mitogen-Activated Protein Kinase
miR: MicroRNA
MMPs: Matrix Metalloproteinases
mTORC1: Mechanistic Target Of Rapamycin Complex 1
NF-κB: Nuclear Factor Kappa-B
PDGF: Platelet-Derived Growth Factor
PDGFRα: Platelet-Derived Growth Factor Receptor Alpha
PDLLA: Poly-D, L-Lactic Acid
PI3K: Phosphoinositide 3-Kinase
PLLA: Poly-L-Lactic Acid
PRP: Platelet-Rich Plasma
RAGE: Receptor for Advanced Glycation End Products
ROS: Reactive Oxygen Species
SASP: Senescence-Associated Secretory Phenotype
SEM: Skin Extracellular Microenvironment
Smad: Sma- and Mad-Related Proteins
STAT: Signal Transducer and Activator of Transcription
TAZ: Transcriptional Co-activator with PDZ-Binding Motif
TEWL: Transepidermal Water Loss
TGF-β: Transforming Growth Factor-Β
TIMPs: Tissue Inhibitors of Metalloproteinases
TLR: Toll-Like Receptor
TNF-α: Tumor Necrosis Factor-Α
VDR: Vitamin D Receptor
VEGF: Vascular Endothelial Growth Factor
YAP: Yes-Associated Protein

## References

[1] Fallacara A., Baldini E., Manfredini S., Vertuani S. (2018). Hyaluronic acid in the third millennium.. Polymers.

[2] Buckley C., Murphy E.J., Montgomery T.R., Major I. (2022). Hyaluronic acid: A review of the drug delivery capabilities of this naturally occurring polysaccharide.. Polymers.

[3] Chen F., Guo X., Wu Y. (2023). Skin antiaging effects of a multiple mechanisms hyaluronan complex.. Skin Res. Technol..

[4] Fan Y., Choi T.H., Chung J.H., Jeon Y.K., Kim S. (2019). Hyaluronic acid-cross-linked filler stimulates collagen type 1 and elastic fiber synthesis in skin through the TGF-β/Smad signaling pathway in a nude mouse model.. J. Plast. Reconstr. Aesthet. Surg..

[5] Cortes-Medina M., Bushman A.R., Beshay P.E., Adorno J.J., Menyhert M.M., Hildebrand R.M., Agarwal S.S., Avendano A., Song J.W. (2023). Chondroitin sulfate, dermatan sulfate, and hyaluronic acid differentially modify the biophysical properties of collagen-based hydrogels.. bioRxiv.

[6] Wang F., Chang H.M., Yi Y., Li H., Leung P.C.K. (2019). TGF-β1 promotes hyaluronan synthesis by upregulating hyaluronan synthase 2 expression in human granulosa-lutein cells.. Cell. Signal..

[7] Huang J., Heng S., Zhang W., Liu Y., Xia T., Ji C., Zhang L. (2022). Dermal extracellular matrix molecules in skin development, homeostasis, wound regeneration and diseases.. Semin. Cell Dev. Biol..

[8] Jiao Q., Zhi L., You B., Wang G., Wu N., Jia Y. (2024). Skin homeostasis: Mechanism and influencing factors.. J. Cosmet. Dermatol..

[9] Holmes D.F., Lu Y., Starborg T., Kadler K.E. (2018). Collagen fibril assembly and function.. Curr. Top. Dev. Biol..

[10] Roig-Rosello E., Rousselle P. (2020). The human epidermal basement membrane: A shaped and cell instructive platform that aging slowly alters.. Biomolecules.

[11] Uno E., Kim F., Yoshino M., Sato Y., Hashimoto M., Watanabe K., Mizukami Y., Muto J. (2025). Targeting inflammatory macrophages with hyaluronan tetrasaccharide: Effects on fibroblast collagen degradation and synthesis.. Front. Immunol..

[12] Romani P., Valcarcel-Jimenez L., Frezza C., Dupont S. (2021). Crosstalk between mechanotransduction and metabolism.. Nat. Rev. Mol. Cell Biol..

[13] Garantziotis S., Savani R.C. (2019). Hyaluronan biology: A complex balancing act of structure, function, location and context.. Matrix Biol..

[14] Gillesberg F.S., Pehrsson M., Bay-Jensen A.C., Frederiksen P., Karsdal M., Deleuran B.W., Kragstrup T.W., Kubo S., Tanaka Y., Mortensen J.H. (2025). Regulation of fibronectin and collagens type I, III and VI by TNF-α, TGF-β, IL-13, and tofacitinib.. Sci. Rep..

[15] Li Q., Fang L., Chen J., Zhou S., Zhou K., Cheng F., Cen Y., Qing Y., Wu J. (2021). Exosomal MicroRNA-21 promotes keloid fibroblast proliferation and collagen production by inhibiting Smad7.. J. Burn Care Res..

[16] Philippeos C., Telerman S.B., Oulès B., Pisco A.O., Shaw T.J., Elgueta R., Lombardi G., Driskell R.R., Soldin M., Lynch M.D., Watt F.M. (2018). Spatial and single-cell transcriptional profiling identifies functionally distinct human dermal fibroblast subpopulations.. J. Invest. Dermatol..

[17] Driskell R.R., Watt F.M. (2015). Understanding fibroblast heterogeneity in the skin.. Trends Cell Biol..

[18] Ganier C., Rognoni E., Goss G., Lynch M., Watt F.M. (2022). Fibroblast heterogeneity in healthy and wounded skin.. Cold Spring Harb. Perspect. Biol..

[19] Dupont S. (2016). Role of YAP/TAZ in cell-matrix adhesion-mediated signalling and mechanotransduction.. Exp. Cell Res..

[20] Lim C.H., Ito M. (2022). Niche stiffness regulates stem cell aging.. Nat. Aging.

[21] Tavianatou A.G., Caon I., Franchi M., Piperigkou Z., Galesso D., Karamanos N.K. (2019). Hyaluronan: Molecular size‐dependent signaling and biological functions in inflammation and cancer.. FEBS J..

[22] Chen A., Huang W., Wu L., An Y., Xuan T., He H., Ye M., Qi L., Wu J. (2020). Bioactive ECM mimic hyaluronic acid dressing via sustained releasing of bFGF for enhancing skin wound healing.. ACS Appl. Bio Mater..

[23] Kay E.J., Koulouras G., Zanivan S. (2021). Regulation of extracellular matrix production in activated fibroblasts: Roles of amino acid metabolism in collagen synthesis.. Front. Oncol..

[24] Walma D.A.C., Yamada K.M. (2020). The extracellular matrix in development.. Development.

[25] Terazawa S., Nakajima H., Tobita K., Imokawa G. (2015). The decreased secretion of hyaluronan by older human fibroblasts under physiological conditions is mainly associated with the down-regulated expression of hyaluronan synthases but not with the expression levels of hyaluronidases.. Cytotechnology.

[26] Khalid K.A., Nawi A.F.M., Zulkifli N., Barkat M.A., Hadi H. (2022). Aging and wound healing of the skin: A review of clinical and pathophysiological hallmarks.. Life.

[27] Wang S.T., Neo B.H., Betts R.J. (2021). Glycosaminoglycans: Sweet as sugar targets for topical skin anti-aging.. Clin. Cosmet. Investig. Dermatol..

[28] Huang S., Strange A., Maeva A., Siddiqui S., Bastien P., Aguayo S., Vaez M., Montagu-Pollock H., Ghibaudo M., Potter A., Pageon H., Bozec L. (2023). Quantitative nanohistology of aging dermal collagen.. Front. Aging.

[29] He T., Fisher G.J., Kim A.J., Quan T. (2023). Age-related changes in dermal collagen physical properties in human skin.. PLoS One.

[30] Vidović T., Ewald C.Y. (2022). Longevity-promoting pathways and transcription factors respond to and control extracellular matrix dynamics during aging and disease.. Front. Aging.

[31] Chen C., Zhang J.Q., Li L., Guo M., He Y., Dong Y., Meng H., Yi F. (2022). Advanced glycation end products in the skin: Molecular mechanisms, methods of measurement, and inhibitory pathways.. Front. Med..

[32] Birch J., Gil J. (2020). Senescence and the SASP: Many therapeutic avenues.. Genes Dev..

[33] Yoon J.E., Kim Y., Kwon S., Kim M., Kim Y.H., Kim J.H., Park T.J., Kang H.Y. (2018). Senescent fibroblasts drive ageing pigmentation: A potential therapeutic target for senile lentigo.. Theranostics.

[34] Shimizu Y., Koguchi-Yoshioka H., Kubo T., Matsumura Y., Matsuda S., Tanemura A., Nomori M., Otani N., Taminato M., Tomita K., Kubo T., Fujimoto M., Watanabe R. (2025). Immunoregulatory microenvironment in the elderly skin.. JID Innov..

[35] Deng Z., Fan T., Xiao C., Tian H., Zheng Y., Li C., He J. (2024). TGF-β signaling in health, disease and therapeutics.. Signal Transduct. Target. Ther..

[36] van den Akker G.G., van Beuningen H.M., Vitters E.L., Koenders M.I., van de Loo F.A., van Lent P.L., Blaney Davidson E.N., van der Kraan P.M. (2017). Interleukin 1 β-induced SMAD2/3 linker modifications are TAK1 dependent and delay TGFβ signaling in primary human mesenchymal stem cells.. Cell. Signal..

[37] Zhang J., Yu H., Man M.Q., Hu L. (2024). Aging in the dermis: Fibroblast senescence and its significance.. Aging Cell.

[38] Chu C.Q., Quan T. (2024). Fibroblast Yap/Taz signaling in extracellular matrix homeostasis and tissue fibrosis.. J. Clin. Med..

[39] Ye H., Zhang R., Zhang C., Xia Y., Jin L. (2024). Advances in hyaluronic acid: Bioactivity, complexed biomaterials and biological application: A review.. Asian J. Surg..

[40] Senatus L.M., Schmidt A.M. (2017). The AGE-RAGE axis: Implications for age-associated arterial diseases.. Front. Genet..

[41] Shin J.W., Kwon S.H., Choi J.Y., Na J.I., Huh C.H., Choi H.R., Park K.C. (2019). Molecular mechanisms of dermal aging and antiaging approaches.. Int. J. Mol. Sci..

[42] Berdiaki A., Neagu M., Spyridaki I., Kuskov A., Perez S., Nikitovic D. (2023). Hyaluronan and reactive oxygen species signaling—Novel cues from the matrix?. Antioxidants.

[43] Montanari E., Zoratto N., Mosca L., Cervoni L., Lallana E., Angelini R., Matassa R., Coviello T., Di Meo C., Matricardi P. (2019). Halting hyaluronidase activity with hyaluronan-based nanohydrogels: Development of versatile injectable formulations.. Carbohydr. Polym..

[44] Duteil L., Queille-Roussel C., Issa H., Sukmansaya N., Murray J., Fanian F. (2023). The effects of a non-crossed-linked hyaluronic acid gel on the aging signs of the face versus normal saline: A randomized, double-blind, placebo-controlled, split-faced study.. J. Clin. Aesthet. Dermatol..

[45] Wang S., Zhu Z., Shih Y., Zhang Y., Liao Y., Chen X. (2025). Dose-dependent effects of cross-linked hyaluronic acid through intradermal injection on female facial photoaging: A prospective study.. Aesthet. Surg. J. Open Forum.

[46] Wu X., Wang B., Liao Y., Li X., Chen J., Zhao L. (2025). Low-crosslinked hyaluronic acid injections in the superficial fat layer for facial rejuvenation in Chinese patients: A retrospective clinical study.. Cureus.

[47] Jiang Y., Jiang Y., Gao Y., Yan L., Zhao D. (2025). The comparative study of the effects of botulinum toxin micro-droplet injection combined with micro-crosslinked sodium hyaluronate gel or focused ultrasound technology in facial rejuvenation treatment.. Aesthetic Plast. Surg..

[48] Karna E., Szoka L., Huynh T.Y.L., Palka J.A. (2020). Proline-dependent regulation of collagen metabolism.. Cell. Mol. Life Sci..

[49] Krymchenko R., Coşar Kutluoğlu G., van Hout N., Manikowski D., Doberenz C., van Kuppevelt T.H., Daamen W.F. (2024). Elastogenesis in focus: Navigating elastic fibers synthesis for advanced dermal biomaterial formulation.. Adv. Healthc. Mater..

[50] Skalny A.V., Aschner M., Silina E.V., Stupin V.A., Zaitsev O.N., Sotnikova T.I., Tazina S.I., Zhang F., Guo X., Tinkov A.A. (2023). The role of trace elements and minerals in osteoporosis: A review of epidemiological and laboratory findings.. Biomolecules.

[51] Pickart L., Margolina A. (2018). Regenerative and protective actions of the GHK-Cu peptide in the light of the new gene data.. Int. J. Mol. Sci..

[52] Gorantla K.R., Krishnan A., Waheed S.O., Varghese A., DiCastri I., LaRouche C., Paik M., Fields G.B., Karabencheva-Christova T.G. (2024). Novel insights into the catalytic mechanism of collagenolysis by Zn(II)-dependent matrix metalloproteinase-1.. Biochemistry.

[53] Kechichian E., Ezzedine K. (2018). Vitamin D and the skin: An update for dermatologists.. Am. J. Clin. Dermatol..

[54] Scarano A., Qorri E., Sbarbati A., Gehrke S.A., Frisone A., Amuso D., Tari S.R. (2024). The efficacy of hyaluronic acid fragments with amino acid in combating facial skin aging: An ultrasound and histological study.. J. Ultrasound.

[55] Svolacchia F., Svolacchia L., Marchetti M., Prisco C., Inchingolo F., Amuso D., Giuzio F., Scarano A. (2023). Evaluation of the efficacy and safety of hyaluronic acid and supplemented with amino acids, and glutathione or colin, for the prevention and treatment of wrinkles on the face, neck, décolleté and hands.. Eur. Rev. Med. Pharmacol. Sci..

[56] Fasola E., Nobile V. (2024). Low molecular weight hyaluronic acid added to six specific amino acids in the treatment of Striae Alba (SA): An observational study.. Aesthetic Plast. Surg..

[57] Kabakci A.G., Cengizler Ç., Eren D., Bozkir M.G. (2024). Morphometric analysis of the effects of high molecular weight sodium hyaluronate and amino acids mixture on face rejuvenation.. J. Cosmet. Dermatol..

[58] Pullar J., Carr A., Vissers M. (2017). The roles of vitamin C in skin health.. Nutrients.

[59] Phoebe L.K.W., Lee K.W.A., Chan L.K.W., Hung L.C., Wu R., Wong S., Wan J., Yi K.H. (2024). Use of platelet rich plasma for skin rejuvenation.. Skin Res. Technol..

[60] Cameli N., Mariano M., Cordone I., Abril E., Masi S., Foddai M.L. (2017). Autologous pure platelet-rich plasma dermal injections for facial skin rejuvenation: Clinical, instrumental, and flow cytometry assessment.. Dermatol. Surg..

[61] Shen J., Yu X., Zhuang Y., Kong Y., Zhao J., Yao Z. (2024). Clinical efficacy and safety of targeted injection of PRP with skin booster in the treatment of aging face.. Biotechnol. Genet. Eng. Rev..

[62] Cecerska-Heryć E., Goszka M., Serwin N., Roszak M., Grygorcewicz B., Heryć R., Dołęgowska B. (2022). Applications of the regenerative capacity of platelets in modern medicine.. Cytokine Growth Factor Rev..

[63] Oh S., Lee J.H., Kim H.M., Batsukh S., Sung M.J., Lim T.H., Lee M.H., Son K.H., Byun K. (2023). Poly-L-lactic acid fillers improved dermal collagen synthesis by modulating M2 macrophage polarization in aged animal skin.. Cells.

[64] Kim S.A., Kim H.S., Jung J.W., Suh S.I., Ryoo Y.W. (2019). Poly-L-lactic acid increases collagen gene expression and synthesis in cultured dermal fibroblast (Hs68) through the p38 MAPK pathway.. Ann. Dermatol..

[65] Amiri M., Meçani R., Niehot C.D., Phillips T., Kolb J., Daughtry H., Muka T. (2023). Skin regeneration-related mechanisms of Calcium Hydroxylapatite (CaHA): A systematic review.. Front. Med..

[66] Oh S., Seo S.B., Kim G., Batsukh S., Park C.H., Son K.H., Byun K. (2023). Poly-D,L-lactic acid filler increases extracellular matrix by modulating macrophages and adipose-derived stem cells in aged animal skin.. Antioxidants.

[67] Inacio P.A.Q., Chaluppe F.A., Aguiar G.F., Coelho C.F., Vieira R.P. (2024). Effects of hydrolyzed collagen as a dietary supplement on fibroblast activation: A systematic review.. Nutrients.

[68] Brandao-Rangel M.A.R., Oliveira C.R., da Silva Olímpio F.R., Aimbire F., Mateus-Silva J.R., Chaluppe F.A., Vieira R.P. (2022). Hydrolyzed collagen induces an anti-inflammatory response that induces proliferation of skin fibroblast and keratinocytes.. Nutrients.

[69] Zhou R., Yu M. (2025). The effect of local hyaluronic acid injection on skin aging: A systematic review and meta‐analysis.. J. Cosmet. Dermatol..

[70] Albouy M., Santos M.D., Laho K., Couchourel D., Tranchepain F. (2025). In vitro evaluation of the effects of multipoint hyaluronic acid‐based intradermal fillers on skin quality using a novel 3D reconstructed skin aging model.. J. Cosmet. Dermatol..

[71] Tang Z., Liu Z., Zhang Y., Luo S., Xu Y., Ren L. (2024). Functional hyaluronic acid microneedles for skin photoaging based on collagen induction and oxidative stress regulation strategies.. Int. J. Biol. Macromol..

[72] Boca A., Fanian F., Smit R., Redaelli A., Goorochurn R., Issa H., Sukmanskaya N., Philippon V., Dell’ Avanzato R. (2024). Evaluation of the performance and safety of a new micro‐needle technology in comparison with the classic needle on the antiaging effects of a biorevitalizing solution: A randomized split face/neck study.. J. Cosmet. Dermatol..

[73] Ye W., Liu T., Zhang W., Peng S., Liao Y. (2026). Assessment of subepidermal low-echogenic band via high-frequency ultrasound for evaluating the efficacy of platelet-rich plasma injection in treating facial skin photoaging: A case series.. J. Cosmet. Dermatol..

[74] Siquier-Dameto G., Boadas-Vaello P., Verdú E. (2024). Intradermal treatment with a hyaluronic acid complex supplemented with amino acids and antioxidant vitamins improves cutaneous hydration and viscoelasticity in healthy subjects.. Antioxidants.

[75] Tedesco L., Rossi F., Ruocco C., Ragni M., Carruba M.O., Valerio A., Nisoli E. (2022). A designer mixture of six amino acids promotes the extracellular matrix gene expression in cultured human fibroblasts.. Biosci. Biotechnol. Biochem..

[76] Sun Y., Zhang S., Shen Y., Lu H., Zhao X., Wang X., Wang Y., Wang T., Liu B., Yao L., Wen J. (2024). Therapeutic application of mesenchymal stem cell-derived exosomes in skin wound healing.. Front. Bioeng. Biotechnol..

[77] Zhu Y., Yang H., Xue Z., Tang H., Chen X., Liao Y. (2025). Mesenchymal stem cells-derived small extracellular vesicles and apoptotic extracellular vesicles for wound healing and skin regeneration: A systematic review and meta-analysis of preclinical studies.. J. Transl. Med..

[78] Yan L., Fan D., Yang J., Wang J., Hu X., Zhang X., Huang Y., Wang H., Yin W., Cai X., Shang R., Huang C., Luo G., He W. (2025). Fibroblast exosomes promote wound healing and improve the quality of healed skin via miR-29a-3p-mediated KEAP1/Nrf2 pathway activation.. Burns Trauma.

[79] Zheng Y., Dong X., Wang X., Wang J., Chen S., He Y., An J., He L., Zhang Y. (2023). Exosomes derived from adipose tissue-derived mesenchymal stromal cells prevent medication-related osteonecrosis of the jaw through IL-1RA.. Int. J. Mol. Sci..

