# Supplementary material for: The Tripartite Regulatory Framework of the Skin Extracellular Microenvironment and Related Anti-aging Strategies
Source: Mini Rev Med Chem. 2026 Apr 20;26(11):771–81. doi: 10.2174/0113895575436479260306062725 (PMC13555759; doi:10.2174/0113895575436479260306062725)
Supplement: Supplementary file 1 [file MRMC-26-11-771_SD1.pdf]

Supplementary Material

The Tripartite Regulatory Framework of the Skin Extracellular Microenvironment and Related Anti-aging Strategies

Liangsen Zhao[1] and Yong Liao[1,*]

[1]*Department of Medicine, Bloomage Biotechnology Corporation Limited, Beijing 100000, China*

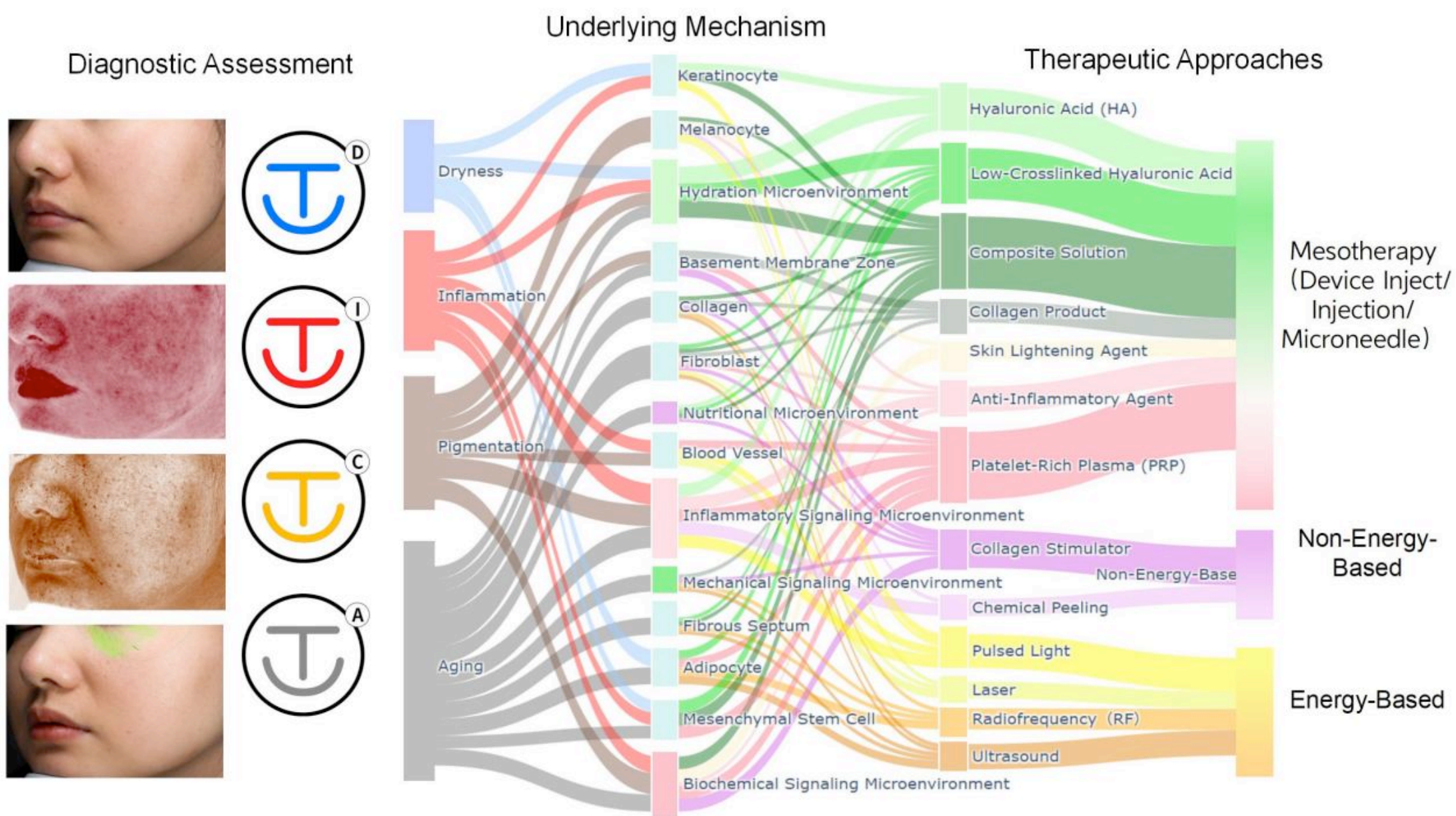

Supplementary Figure S1. Clinical diagnosis–mechanism–treatment mapping within the skin extracellular microenvironment (SEM). *(A higher resolution / colour version of this figure is available in the electronic copy of the article).*

This diagram integrates clinical phenotypes, underlying SEM mechanisms, and corresponding therapeutic strategies into a unified translational framework. On the left, representative clinical manifestations (*e.g.*, dryness, inflammation, pigmentation, and aging-related texture changes) reflect diagnostic outcomes commonly encountered in aesthetic practice. These phenotypes are mapped to their underlying mechanisms within the SEM (center), including hydration, nutritional, inflammatory, biochemical, and mechanical signaling microenvironments, as well as their associated cellular components (keratinocytes, fibroblasts, adipocytes, and vascular units). Building upon the structural–functional organization of the SEM illustrated in Fig. (**1**) and the degenerative alterations and intervention targets summarized in Fig. (**2**), the right panel translates these mechanisms into clinically actionable treatment strategies. These include mesotherapy-based approaches (*e.g.*, hyaluronic acid, low-crosslinked HA, composite solutions, PRP), non-energy-based modalities (*e.g.*, collagen stimulators, chemical peeling), and energy-based devices (*e.g.*, laser, radiofrequency, ultrasound).

DISCLAIMER: The above article has been published, as is, ahead-of-print, to provide early visibility but is not the final version. Major publication processes like copyediting, proofing, typesetting and further review are still to be done and may lead to changes in the final published version, if it is eventually published. All legal disclaimers that apply to the final published article also apply to this ahead-of-print version.
